# Paclitaxel treatment enhances lymphatic metastasis of B16F10 melanoma cells via CCL21/CCR7 axis

**DOI:** 10.7150/ijbs.67138

**Published:** 2022-01-24

**Authors:** Li Zhang, Linyu Zhu, Xiaohan Yao, Xiaohan Lou, Jiajia Wan, Xixi Duan, Longze Pan, Anqi Li, Zhuoyu Gu, Ming Wang, Fazhan Wang, Zhihai Qin

**Affiliations:** 1 Medical Research Center, The First Affiliated Hospital of Zhengzhou University, Zhengzhou University, Zhengzhou, 450052, Henan, China.; 2 Academy of Medical Sciences, Zhengzhou University, Zhengzhou, 450052, Henan, China.; 3 Institute of Biophysics, Chinese Academy of Sciences, Beijing 100101, China.

**Keywords:** Chemotherapy, Lymphatic metastasis, CCL21/CCR7 axis, B16F10, Cell migration

## Abstract

Chemotherapeutic drugs have been successfully used to treat several cancers, including melanoma. However, metastasis occasionally occurs after chemotherapy. Here, we reported that paclitaxel (PTX) treatment for B16F10 tumour in mice led to an enhanced lymphatic metastasis of the melanoma cells, although a significant inhibition of tumour growth at the injection site was observed. Further study demonstrated that PTX upregulated the expression of C-C chemokine receptor type 7 (CCR7) in B16F10 cells, enhancing their migration through the activation of JNK and p38 signalling pathways. Loss of CCR7 or blockade of C-C motif chemokine ligand 21 (CCL21)/CCR7 axis abolished the pro-migration effect of PTX on B16F10 melanoma cells. Importantly, combination of PTX and CCR7 mAb could simultaneously delay the tumour growth and reduce the lymphatic metastasis in B16F10 melanoma. The blockade of CCL21/CCR7 axis may collectively serve as a strategy for lymphatic metastasis in some melanoma after chemotherapy.

## Introduction

Chemotherapy remains one of the major strategies for treating malignant tumours, although there are many clinical strategies for tumour treatment 1. Chemotherapeutic drugs delay tumour progression mainly by killing tumour cells through a cytotoxic effect 2. PTX, a classic taxane chemotherapeutic drug, has been used to treat many malignant cancers, including breast cancer and melanoma 3, 4. However, it has been reported that PTX could promote lymphatic metastasis of breast cancer in some cases, besides inhibiting tumour growth 4, 5. Lymphatic metastasis is a significant basis for determining clinical treatment and predicting the prognosis of patients with tumours after treatment 6, 7.

Tumour metastasis is attributable to more than 90% of tumour-related deaths 8. Tumour cells metastasise *in vivo* in several ways, including directly invading surrounding tissues, transferring to distant organs through blood (vascular metastasis), and reaching lymph nodes and distant organs through the lymphatic system (lymphatic metastasis) 9. Lymphatic metastasis is one of the predominant metastatic pathways for most tumour metastasis 10 and occurs early during tumour development 11. According to the previous reports, PTX could activate toll-like receptor-4 (TLR4) to enhance systemic inflammation and promote lymphangiogenesis and breast cancer lymphatic metastasis 12. In addition, chemotherapeutic drugs mediate a higher permeability of lymphatic endothelial cells to tumour cells by inducing autophagy in lymphatic endothelial cells, which significantly enhances breast cancer metastasis to lymph nodes 13. However, not much attention is paid to the lymphatic metastasis of melanoma, a malignant tumour prone to lymphatic metastasis, after chemotherapy.

Recent studies have shown that the crosstalk between chemokines and their receptors play a critical role in lymphatic metastasis 14. CCL21/CCR7 signalling axis was initially studied in immune cells, although further investigations demonstrated its crucial role in cancer cells for lymphatic metastasis 15, 16. CCL21, secreted mainly by lymphatic endothelial cells, can recruit CCR7-expressed cells to lymphatic vessels 17. CCR7, expressed primarily to immune cells, such as dendritic cells (DCs), T cells, and B cells, mediates immune cells homing to lymph nodes 18. According to recent studies, CCR7 is highly expressed in many cancers, including breast cancer 19, oesophageal squamous cell carcinoma 15 and melanoma 20. Overexpression of CCR7 is associated with tumour lymphatic metastasis by lymphangiogenesis 21 and epithelial-mesenchymal transition 22, 23. Activation of CCL21/CCR7 promoted proliferation and migration of tumour cells 24. Furthermore, CCL21/CCR7 upregulated MUC1 via the ERK1/2 signalling pathway, which could promote the lymph node metastasis of oesophageal squamous cell carcinoma 15. Collectively, pre-clinical data in mouse models suggested that the pro-metastatic capacity of the CCL21/CCR7 axis may facilitate cancer cells migration from the primary tumour to lymph nodes; however, whether CCL21/CCR7 axis plays a critical role in lymphatic metastasis of melanoma after chemotherapy remains unclear.

The current study investigated whether CCL21/CCR7 axis involves lymphatic metastasis of melanoma after chemotherapy. The B16F10 melanoma lymph node metastasis mouse model was established and treated with PTX. Further, the tumour growth and lymphatic metastasis were evaluated. To further clarify the mechanism of the CCL21/CCR7 axis on lymphatic metastasis of melanoma after PTX treatment, RNA sequencing, quantitative PCR, western blot, and migration experiment were performed. To study whether blocking the CCL21/CCR7 axis can inhibit the lymphatic metastasis of melanoma after PTX treatment, CCR7 mAb was applied to block the CCL21/CCR7 signalling pathway *in vivo*.

## Materials and Methods

### Reagents and antibodies

CCL21 (#250-13) was purchased from PEPROTECH. The ERK inhibitor PD98059 (#HY-12028), JNK inhibitor SP600125 (#HY-12041), and the p38 inhibitor BIRB796 (#HY-10320) were purchased from MedChemExpress. Details of all antibodies are listed in Table S1.

### Cell lines

B16F10 cells were purchased from ATCC. B16F10-mCherry cells are stably transformed B16F10 expressing mCherry protein. Cells were cultured in modified RPMI medium (1640, Hyclone) containing 10% foetal calf serum (FBS, PAN Biotech) and 100 IU/mL penicillin/streptomycin (Gibco). 4T1 cells were purchased from ATCC and were cultured in DMEM/High Glucose medium (Hyclone). All cells were maintained in a humidified atmosphere containing 5% carbon dioxide at 37 °C.

### Animal studies

All experiments were performed using 6-8-week-old wild-type female C57BL/6N mice (Vitonglihua Laboratory Animal Technology Co., Ltd., Beijing). All experimental animal protocols were approved by the Committee of Zhengzhou University on Ethics. To study the effect of PTX on lymphatic metastasis, B16F10-mCherry (5 × 10^5^ cells) melanoma cells were implanted into the right footpad of mice. Mice (n = 6) were intravenously injected with PTX at 16, 18, 20, 22, and 24 days after tumour injection. Tumour volume was measured periodically with a vernier calliper according to the formula 0.5 × lengths × width^2^. Mice were sacrificed 26 days after tumour injection; posterior genicular fossa lymph nodes were removed and embedded in paraffin for paraffin-embedded sections and H&E staining. Tumours were excised and embedded in OCT for frozen sections and immunofluorescence staining.

To study the effect of blocking the CCL21/CCR7 axis on lymphatic metastasis of melanoma after PTX treatment, B16F10-mCherry (5 × 10^5^ cells) melanoma cells were implanted into the right footpad of mice. Mice were randomised into four groups (n = 5) with different treatments as follows: vehicle control (control group), PTX (PTX group), CCR7 monoclonal antibody (CCR7 mAb group), and the combination of PTX and CCR7 mAb (PTX+CCR7 mAb group). Tumour size was monitored every 2 days. At the endpoint on day 22, tumours, sentinel lymph nodes, and major organs were harvested and tested.

### Cytotoxic drug treatment

PTX (MACKIN, #P875571) was dissolved in a solvent mixture of polyethylene castor oil and anhydrous ethanol (aladdin, #E111991) (1:1) 5, 25 to make a stock concentration of 10 mg/mL. The mixture of polyethylene castor oil and anhydrous ethanol (1:1) is highly toxic. In this study, the PTX-containing solvent mixture was diluted 10^5^ times with a cell culture medium before its application in cell studies. The solvent mixture of polyethylene castor oil and anhydrous ethanol (1:1) was used as the vehicle control (no PTX). For *in vitro* experiments, B16F10 cells were treated without or with 12.5, 25, 50, or 100 ng/mL PTX. For animal experiments, mice with tumours were injected with vehicle control or PTX at 2.5, 5, or 10 mg/kg through the tail vein. Cisplatin (DDP) (Solarbio, #D8810) was dissolved in DMSO to obtain a concentration of 25 mg/mL. DDP was added to the cell culture medium to obtain 12.5, 25, 50, and 100 ng/mL concentrations. Doxorubicin hydrochloride (DOX) (CSN pharm, #CSN16255) was dissolved in ddH_2_O at a concentration of 10 mg/mL. The experiments were terminated at 12 or 24 h after adding cytotoxic drugs and vehicle control into the cell culture medium. Mice received a dose of PTX 5 times every 2 days.

### RNA sequencing

The gene expression of B16F10 cells treated with PTX (or vehicle control) was identified using RNA sequencing. In brief, B16F10 cells were treated with 100 ng/mL PTX for 24 h at 37 °C. Further, the RNA samples were extracted and used for RNA sequencing. RNA sequencing was performed by HuaDa Genomic Co. Ltd. (Shenzhen, China) using the Illumina Hiseq 2000 sequencer. The data were analysed using the EdgeR software.

### Small-interference RNAs (siRNAs)

The CCR7-siRNAs were purchased from GenePharma (Shanghai, China). The sequences of siRNAs are listed in Table S2. B16F10 cells (3 ×10^5^ cells/well) were seeded into 6-well plates and were grown to reach a confluency of 50%-70%. Further, the cells were transfected with 50 nM CCR7 siRNA for 8 h using GP-transfect-Mate (GenePharma, #G04008) as per the manufacturer's instructions.

### Isolation of RNA and real-time PCR

Total RNA was extracted from cells with Trizol reagent (Life Technologies). RNA was measured using NanoDrop 2000. Complementary DNA (cDNA) was synthesised from 1 µg of total RNA through reverse transcription with PrimeScript RT Master Mix (Takara, #RR036A). cDNA was analysed using TB Green Premix Ex Taq Ⅱ (Takara, #RR820A) on an ABI PRISM 7300HT Sequence Detection System (Applied Biosystems, Foster City, CA, USA). The relative gene expression was normalised with β-actin for mRNA and was quantified using the 2^-ΔΔCt^ method. The sequences of all primers are listed in Table S3.

### Western blotting

Cell and tissue lysates were obtained in RIPA buffer (Solarbio, #R0020) containing 1% Halt Protease and Phosphatase inhibitor. The protein concentrations were quantified using a BCA Protein Assay Kit (Thermo Scientific, #233225) per the manufacturer's instructions. Proteins (20 μg) were separated by 10% SDS-PAGE and transferred to PVDF membranes. PVDF membranes were stained for 2 min with Fast Green FCF to assess the protein loading. Membranes were blocked with 3% BSA in TBST for 1 h at room temperature and incubated overnight with primary antibodies (anti-CCR7, anti-ERK, anti-phospho-ERK, anti-JNK, anti-phospho-JNK, anti-p38, or anti-phospho-p38) as per the manufacturer's instructions. The membranes were washed with TBST for 8 min for three times. Further, they were incubated with HRP-conjugated anti-rabbit secondary antibodies for 1 h at room temperature. Protein loading was quantified using an anti-β-actin antibody. Proteins were visualised and quantified using the ChemiDoc MP imaging system (Bio-Rad).

### Wound-healing assay

To evaluate the migration ability of B16F10 cells *in vitro*, we performed a wound-healing assay. Cells (3 × 10^5^) were seeded into 6-well plates and incubated overnight at 37 °C. Further, the cells were incubated with RPMI-1640 medium containing 10% FBS with or without PTX (12.5 ng/mL) for 12 h at 37 °C with 5% CO_2_. Cells with or without PTX were seeded into 96-well plates (Corning, #3599) and grown till 100% confluency was obtained. Cells were scratched using a 96-pin wound maker to form a vertical wound in the central area of cells, and the separated cells were carefully washed with serum-free RPMI-1640 medium. Cells were cultured with RPMI-1640 medium containing 4% FBS with or without CCL21 at 37 °C with 5% CO_2_. The wound area was captured with the IncuCyte ZOOM System (Essen BioScience) over 1 day. Cell migration activity was expressed by measuring the distance of the wound gap. The wound-healing experiments were performed in triplicates.

### Immunofluorescence

For immunofluorescence staining of tumour tissues, the harvested tumour tissues were embedded in OCT and subsequently frozen at -80 °C. The tumour tissues were sectioned at 7-μm thickness using a Leica CM1950 Cryostat (Leica Biosystems). The tissue sections were fixed in ice-cold acetone for 10 min, dried overnight in the fume hood, and frozen at -80 °C. Before the experiments, the sections were removed from the -80 °C freezer, brought to room temperature, and washed for 5 min three times with PBS. The sections were fixed in 4% paraformaldehyde for 15 min. After washing three times with PBS, the sections were blocked with 5% BSA for 30 min at room temperature. The samples were incubated overnight with primary antibodies (rabbit anti-CCR7, rat anti-mouse CD31, and rabbit anti-LYVE1) in 5% BSA at 4 °C. The sections were washed for 5 min three times with TBST and incubated with secondary antibodies in 5% BSA for 30 min at room temperature. After washing three times with TBST, nuclei were counterstained with DAPI, and images were recorded using a Perkin Elmer Vectra machine. The negative controls were treated without a primary antibody.

### Statistical analysis

Results were presented as mean ± SEM or ± SD. All statistical analyses were performed using GraphPad Prism. All experiments were performed in triplicates. The statistical analyses were performed using student's* t*-test for analyses between two groups and one-way analysis of variance for analyses of multiple groups. P < 0.05 was considered significant.

## Results

### PTX facilitated the lymphatic metastasis of B16F10 in a popliteal lymph node metastasis mouse model

**Figure 1:**
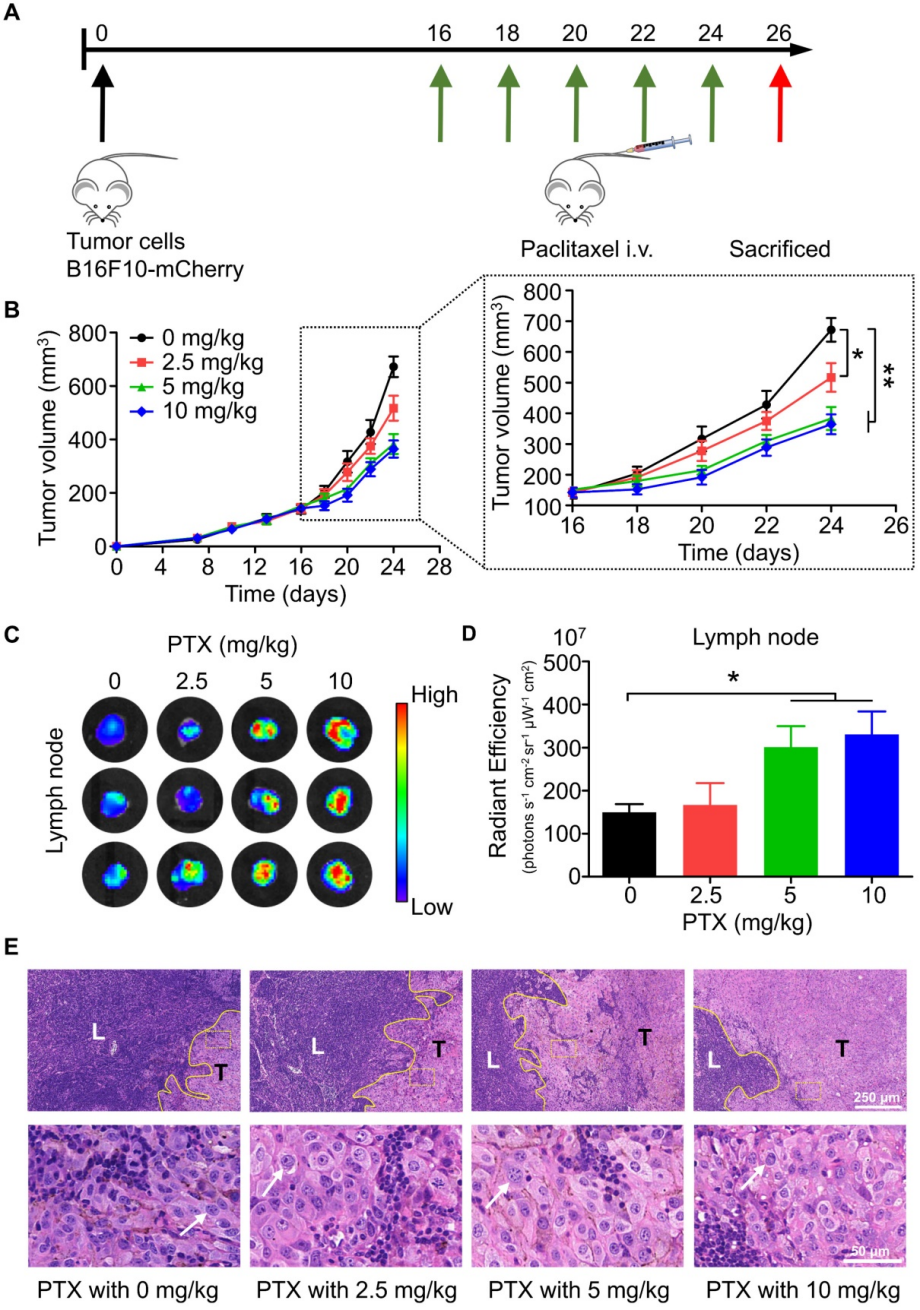
** PTX delayed tumour growth but promoted lymphatic metastasis in B16F10-mCherry-bearing mice. A.** Experimental design and drug scheduling in tumour-bearing mice. **B.** Tumour growth curve with PTX treatments 5 times on days 16, 18, 20, 22, and 24, respectively (n = 6 mice/group). **C.** Bioluminescence imaging of the draining LNs with IVIS at the end of the experiment. **D.** The quantification analysis of the bioluminescence signals of the draining LNs. **E.** Representative haematoxylin/eosin (H&E) images of the draining LN sections of B16F10-mCherry-bearing mice (Scale bars: 250 µm and 50 µm). *P < 0.05; **P < 0.01.

To study the effects of chemotherapy on lymphatic metastasis *in vivo*, we established a popliteal lymph node metastasis model by injecting B16F10-mCherry into the footpad of mice, followed by intravenous injection of PTX (Figure 1A). According to the results, PTX had satisfactory inhibitory activity on the growth of melanoma tumours *in vivo* (Figure 1B). However, mice bearing B16F10-mCherry increased lymphatic metastasis after PTX treatment (Figures 1C-E). Meanwhile, lung metastasis was not observed in PTX-treated mice (Figure S1). To determine whether the PTX-induced B16F10 metastasis was induced by increased lymphatic vessel or blood vessel, we performed immunofluorescence labelling for LYVE1 (a lymphatic vessel marker) and CD31 (a blood vessel marker). No significant difference in lymphatic and blood vessels in tumour tissues was observed after PTX treatment (Figure S2).

### PTX enhanced CCR7 expression in B16F10 *in vivo* and *in vitro*

**Figure 2:**
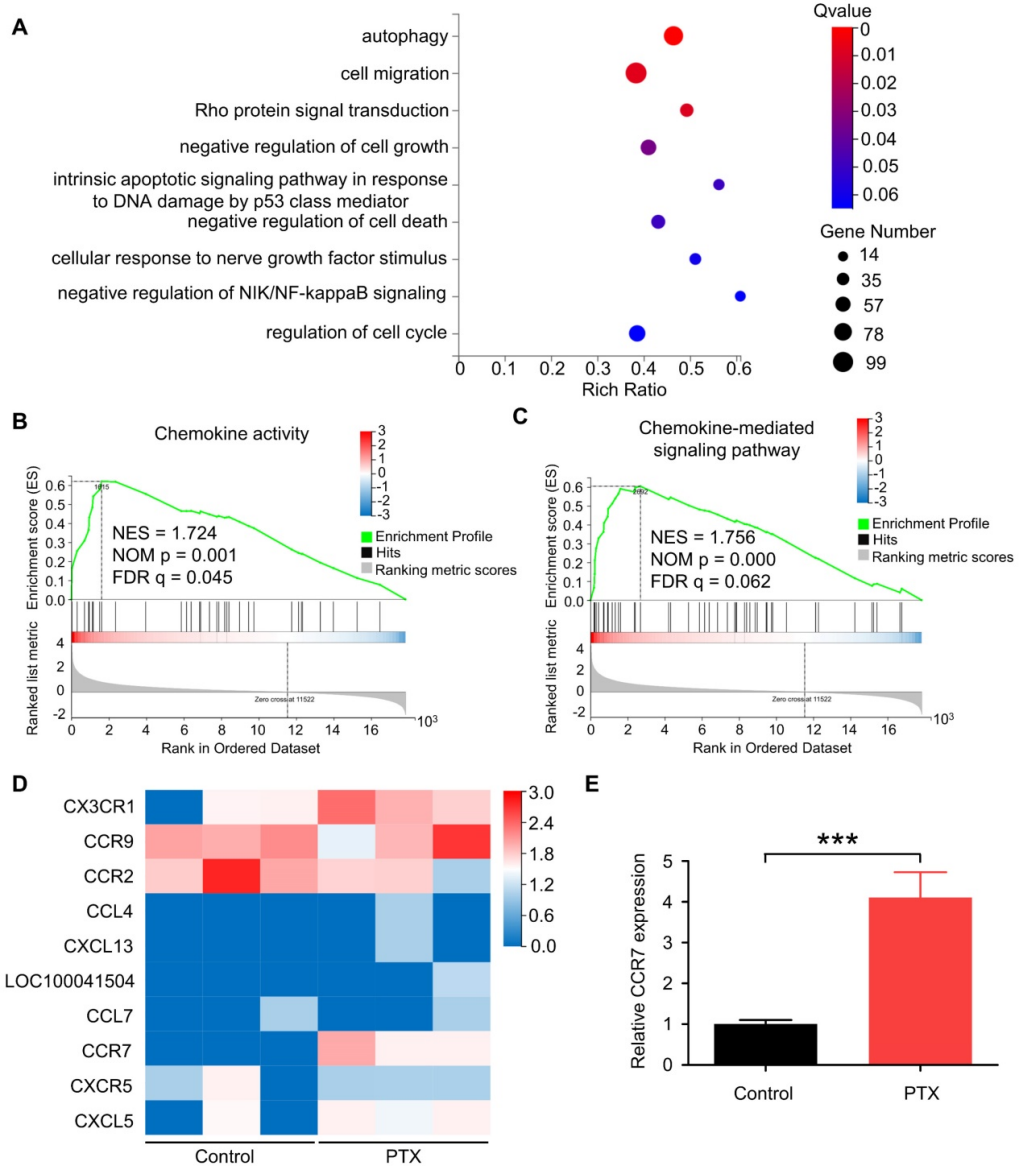
** PTX-promoted tumour metastasis was related to CCR7. A.** GO enrichment analysis of genes in B16F10 cells treated with or without PTX. **B-C.** The plot of gene set enrichment analysis (GESA) using RNA-seq expression profile of B16F10 cells with and without PTX treatment (GO: 0008009, chemokine activity; GO: 0070098, chemokine-mediated signalling pathway). **D.** The expression level of 10 candidate genes in B16F10 cells treated with or without PTX. The heatmap shows the expression of each gene. **E.** RT-qPCR assessed relative CCR7 expression levels in B16F10 cells. ***P < 0.001.

To determine the gene expression levels of B16F10 cells after PTX treatment, we performed RNA sequencing of B16F10 cells treated with or without PTX. According to enrichment analysis of gene ontology (GO), genes were significantly enriched in cell migration (Figure 2A). Recent studies have shown that chemokines and chemokine receptors regulate the migration of various cells 26, 27. Further, we reported cell chemotaxis using RNA-seq GSEA analysis (Figure S3A). Subsequently, we analysed chemokine activity (Figure 2B), chemokine receptor activity (Figure S3B), and chemokine-mediated signalling pathway using RNA-seq GSEA analysis (Figure 2C). Among the altered genes related to the chemokine-mediated signalling pathway, CCR7 was most significantly upregulated in PTX-treated tumour cells (Figure 2D). Further, the mRNA level of *Ccr7* in B16F10 cells was verified after PTX treatment (Figure 2E).

**Figure 3:**
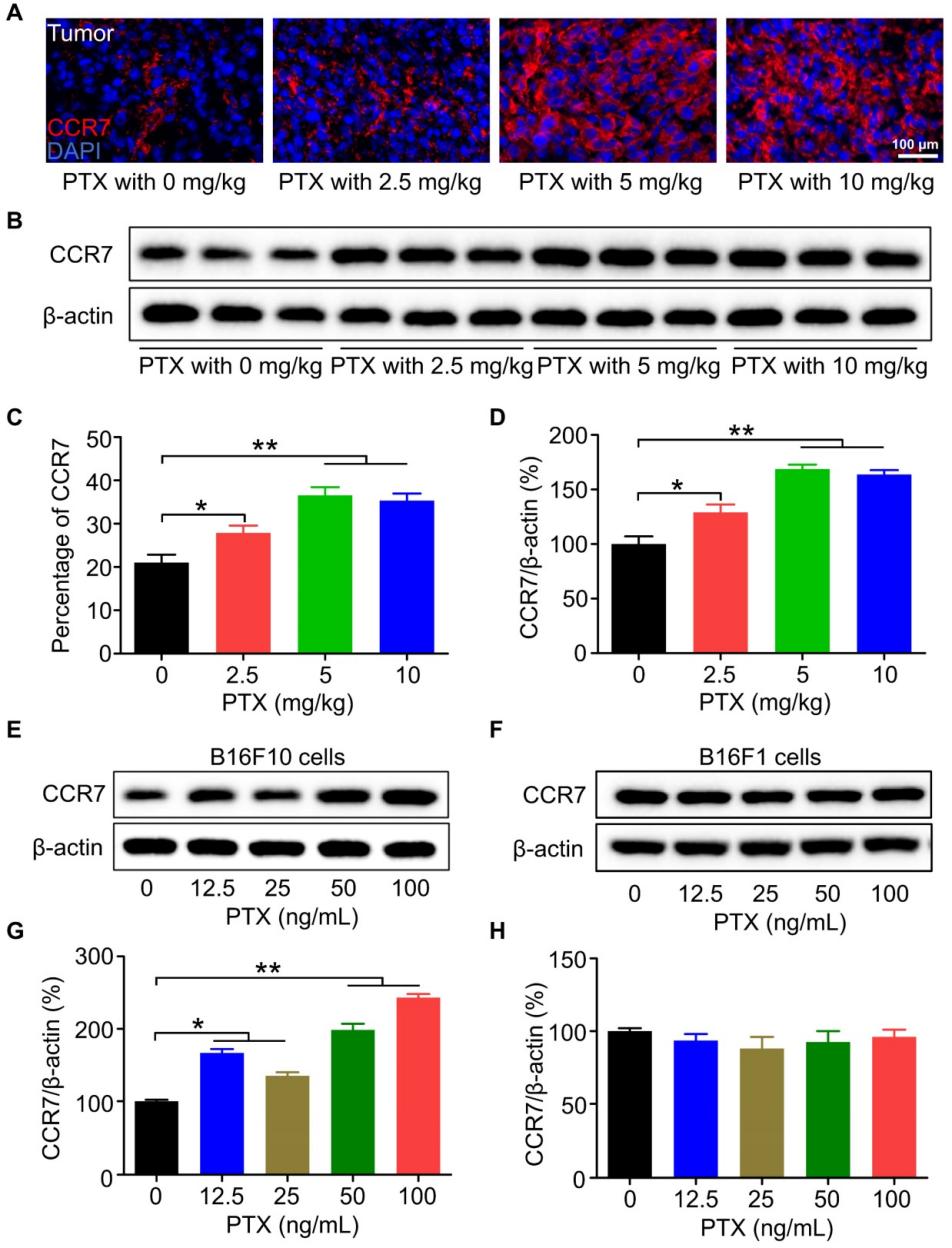
** CCR7 was elevated *in vivo* and *in vitro* after PTX treatment. A.** Immunofluorescence staining of CCR7 in the tumour tissues after PTX treatment (Scale bar: 100 µm, red: CCR7, blue: nucleus). **B.** Western blot analysis of CCR7 expression in the tumour tissues. **C.** The quantification of immunofluorescence staining of CCR7 in the tumour. **D.** The quantification of western blot of CCR7 in the tumour. **E.** Western blot of CCR7 in B16F10 cells with PTX treatment. **F.** Western blot of CCR7 in B16F1 cells with PTX treatment. **G.** The quantification of western blot of CCR7 in B16F10 cells with PTX treatment. **H.** The quantification of western blot of CCR7 in B16F1 cells with PTX treatment. *P < 0.05; **P < 0.01.

The mRNA level of *Ccr7* was significantly increased after PTX treatment; therefore, we next confirmed the increased expression of CCR7 protein by immunofluorescence staining (Figures 3A and C) and western blot analysis (Figures 3B and D). Moreover, the expression of CCR7 was elevated in B16F10 cells treated with PTX *in vitro* compared with that treated with vehicle control (Figures 3E and G). Except for PTX, DDP (Figures S4A and B) but not DOX (Figures S4C and D) could also upregulate the expression of CCR7 in B16F10 cells. However, PTX could not upregulate the expression of CCR7 in 4T1 cells (Figures S4E and F). Furthermore, our results demonstrated that PTX could not upregulate the expression of CCR7 in B16F1 melanoma cells (Figures 3F and H). Overall, these results indicated that the upregulation of CCR7 is dependent on cell types and chemotherapeutic drugs.

### PTX augmented CCL21-induced migration of B16F10

**Figure 4:**
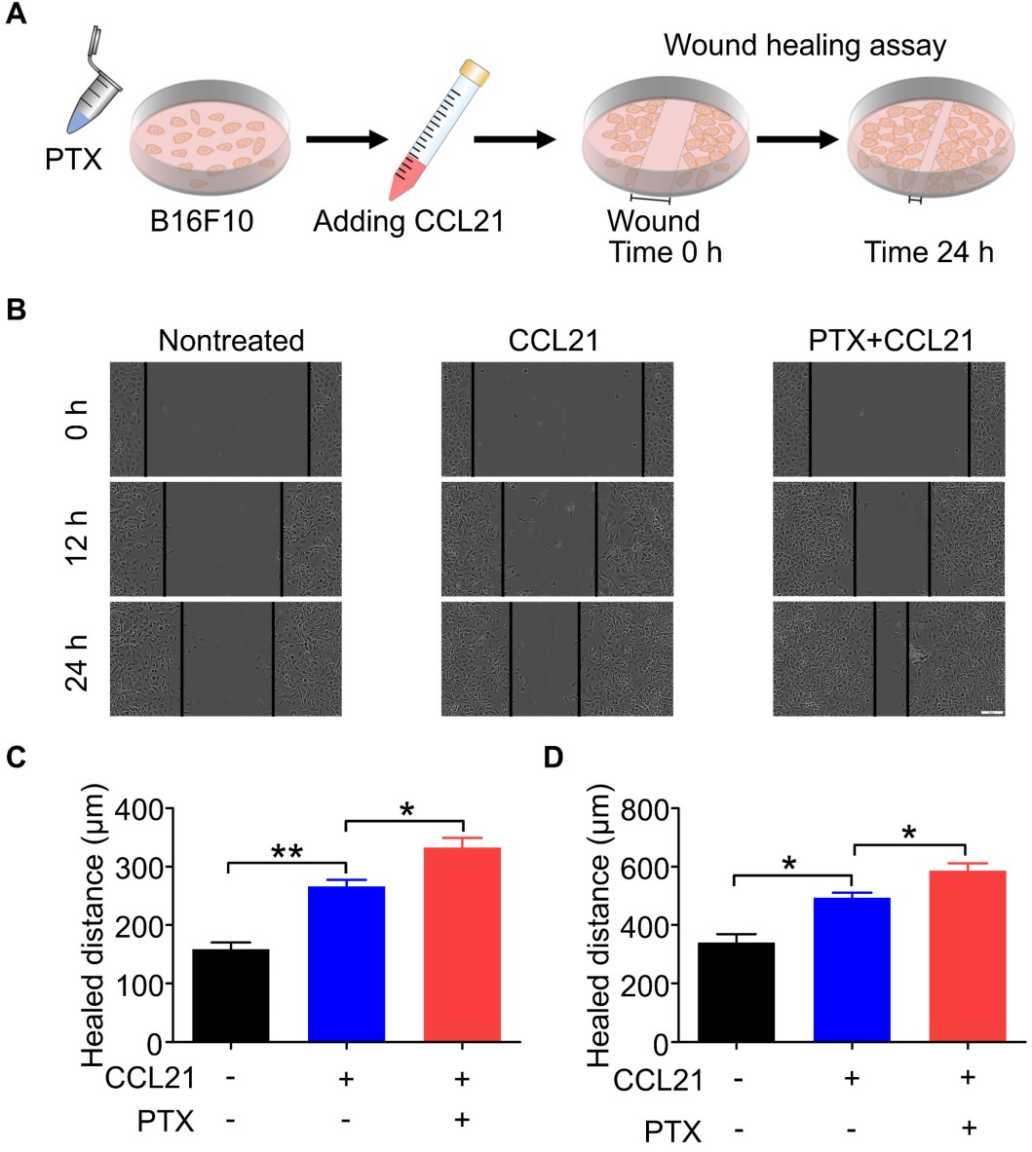
** PTX augmented CCL21-induced migration of B16F10 cells. A.** Experimental scheme of the wound-healing assay for migration evaluation. B16F10 cells were pre-treated with 12.5 ng/mL PTX, and further, the cells were seeded into a 96-well plate. Confluent monolayer of B16F10 cells was scratched and incubated with a culture medium with CCL21. **B.** Images of the wound before and after treatment with PTX (Scale bar: 100 µm). **C.** The quantification of migration distance at 0 and 12 h. **D.** The quantification of migration distance at 0 and 24 h. *P < 0.05; **P < 0.01.

A wound-healing assay was performed to determine the functional role of the CCL21/CCR7 signalling axis in B16F10 cells after PTX treatment. Therefore, the migration and invasion behaviours of B16F10 cells treated with or without PTX were investigated with a wound-healing assay (Figure 4A). Although CCL21 led to an increase in the migration of B16F10 cells, a significant increase in migration was observed in B16F10 cells after PTX treatment compared with that in the nontreated group (Figure 4B). The pre-treatment of B16F10 cells with 12.5 ng/mL PTX led to a significant increase in migration compared to untreated cells (Figures 4C and D). These results indicated that PTX enhanced CCL21-mediated migration of B16F10 cells.

### PTX promoted B16F10 cells migration through the upregulation of CCR7

**Figure 5:**
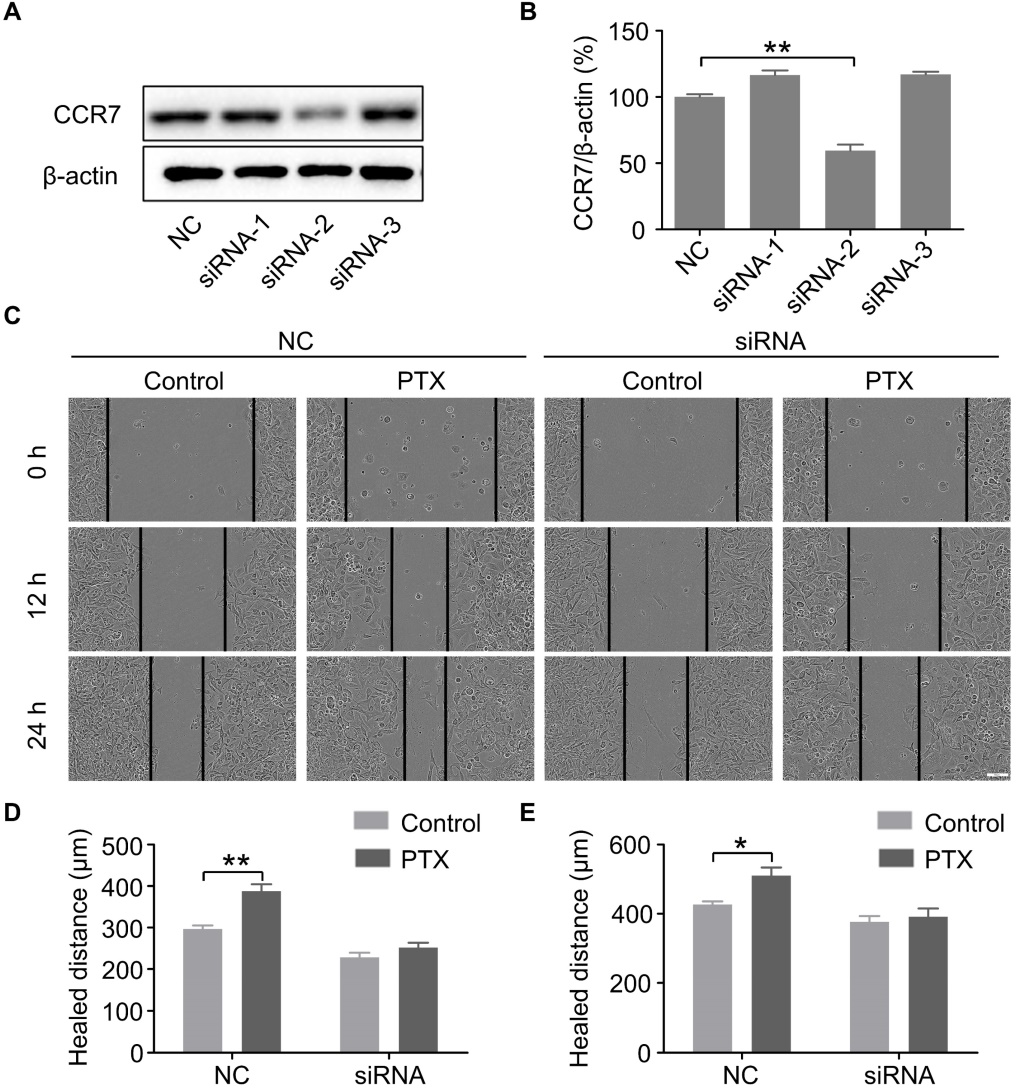
** PTX promoted B16F10 cell migration through CCL21/CCR7 signalling axis. A.** Western blot of CCR7 in B16F10 cells after siRNA treatment.** B.** The quantification of western blot of CCR7 in B16F10 cells after siRNA treatment. **C.** Images of the wound with or without siRNA after PTX treatment (Scale bar: 100 µm).** D.** The quantification of migration distance at 0 and 12 h. **E.** The quantification of migration distance at 0 and 24 h. *P < 0.05; **P < 0.01.

To further verify the above findings, we knocked down the expression of CCR7 using siRNAs (Figures 5A and B). To determine whether PTX promotes B16F10 cell migration through the CCL21/CCR7 signalling axis, we performed a wound-healing assay in B16F10 cells with CCR7 knockdown (Figure 5C). The results revealed that knockdown of CCR7 decreased the migration of B16F10 cells after PTX treatment (Figures 5D and E). It further demonstrated that the progress of tumour metastasis with PTX treatment is associated with CCR7 expression.

### PTX enhanced the activation of ERK, JNK, and p38 in B16F10

**Figure 6:**
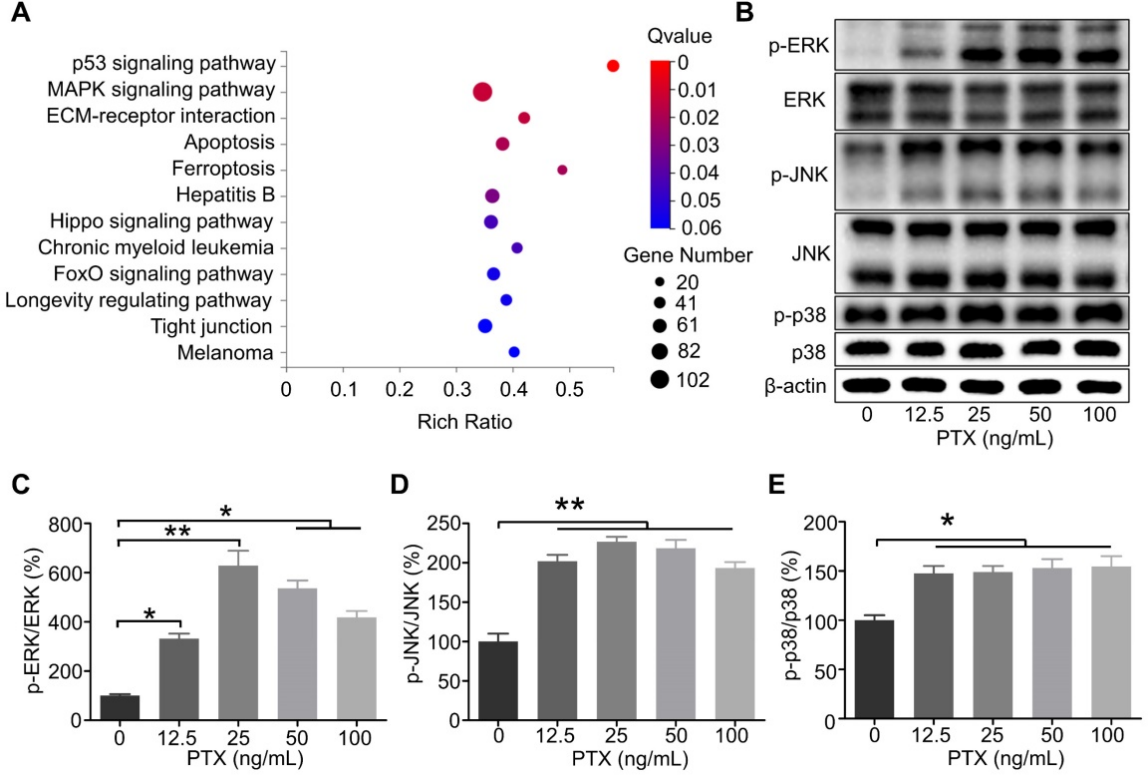
** The phosphorylation level of ERK, JNK, and p38 in B16F10 cells after PTX treatment was enhanced. A.** KEGG enrichment analysis of genes in B16F10 cells treated with or without PTX.** B.** Phosphorylation of ERK, JNK, and p38 in B16F10 cells 24 h after PTX treatment was assessed using western blotting. **C.** The quantification of phosphorylation of ERK in B16F10 cells 24 h after PTX treatment. **D.** The quantification of phosphorylation of JNK in B16F10 cells 24 h after PTX treatment. **E.** The quantification of phosphorylation of p38 in B16F10 cells 24 h after PTX treatment. *P < 0.05; **P < 0.01.

According to the RNA sequencing results, the genes were significantly enriched in the mitogen-activated protein kinase (MAPK) signalling pathway as per the Kyoto Encyclopedia of Genes and Genomes analysis (Figure 6A). It has been reported that the interaction between CCR7 and MAPK plays a vital role in the cell migration and lymphatic metastasis of several tumours. According to studies, CCL21/CCR7 signalling axis can activate MAPK family members, such as ERK, JNK, and p38, to enhance the migration ability of DCs 28. Similarly, CCL21/CCR7 signalling axis may regulate the migration ability of B16F10 by MAPK family members. Therefore, ERK, JNK, and p38 were investigated as potential downstream factors of the CCL21/CCR7 signalling axis during B16F10 cell migration after PTX treatment. As shown in Figures 6B-E, the phosphorylation of ERK, JNK, and p38 was significantly increased in B16F10 cells after PTX treatment for 24 h. Similar results were observed when B16F10 cells were treated with PTX for 12 h (Figure S5).

### CCL21/CCR7 signalling axis promoted B16F10 migration after PTX treatment through activation of JNK and p38

**Figure 7:**
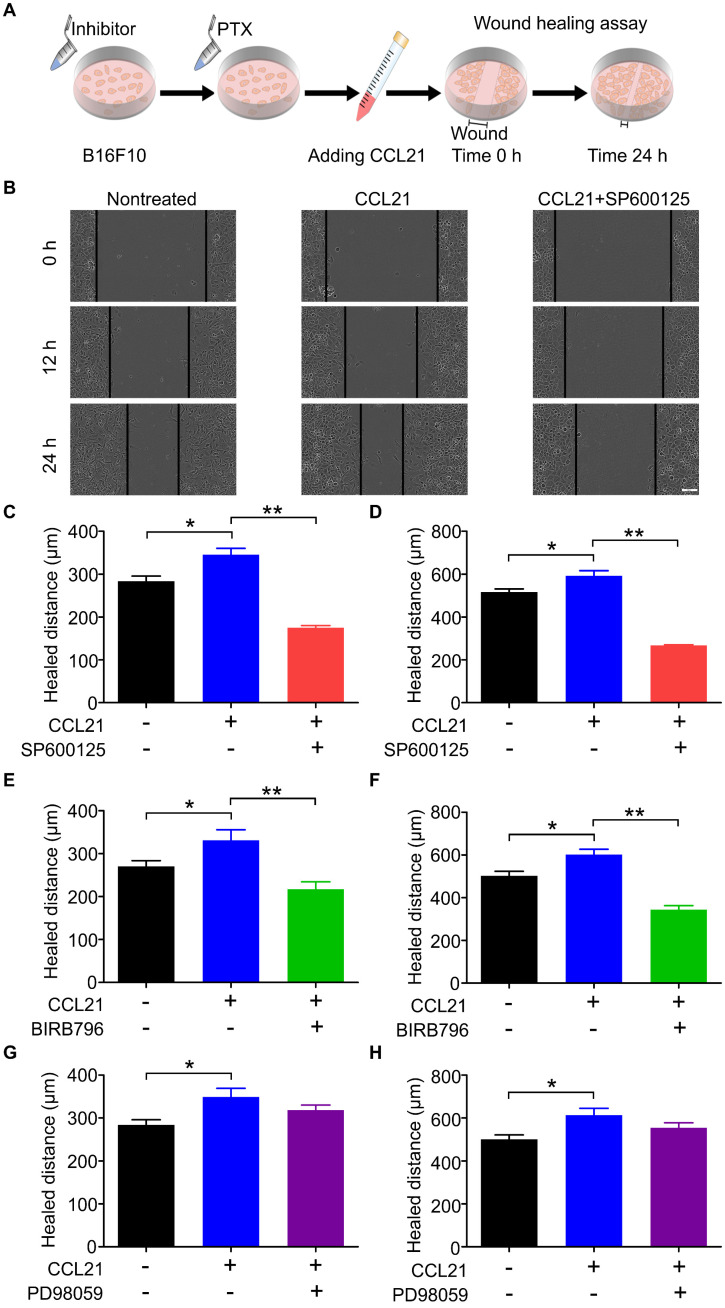
** CCL21/CCR7 signalling-mediated migration of B16F10 cells after PTX treatment was inhibited by blocking JNK and p38. A.** Scheme of the wound-healing assay for migration evaluation. B16F10 cells were first pre-treated with inhibitors of JNK, p38, and ERK 2 h before pre-treatments with PTX for 12 h. **B.** Images of the wound in B16F10 cells pre-treated with JNK inhibitor (SP600125) and PTX (Scale bar: 100 µm). **C.** The quantification of migration distance analysed from panel B. Width of the wound was measured at 0 and 12 h. **D.** The quantification of migration distance was analysed from panel B. The width of the wound was measured at 0 and 24 h. **E.** The quantification of migration distance in B16F10 cells pre-treated with p38 inhibitor (BIRB796). The width of the wound was measured at 0 and 12 h. **F.** The quantification of migration distance in B16F10 cells pre-treated with p38 inhibitor. The width of the wound was measured at 0 and 24 h. **G.** The quantification of migration distance in B16F10 cells pre-treated with ERK inhibitor (PD98059). The width of the wound was measured at 0 and 12 h. **H.** The quantification of migration distance in B16F10 cells pre-treated with ERK inhibitor. The width of the wound was measured at 0 and 24 h. *P < 0.05; **P < 0.01.

Our previous data indicated that the phosphorylation level of ERK, JNK, and p38 was increased in B16F10 cells after PTX treatment. To demonstrate whether the phosphorylation of ERK, JNK, and p38 is related to the migration ability of B16F10 cells after PTX treatment, the inhibitory effects of ERK, JNK, and p38 inhibitors were first confirmed using western blot (Figure S6). In the wound-healing assay (Figure 7A), SP600125 (JNK inhibitor) significantly suppressed CCL21/CCR7-mediated B16F10 cells migration (Figures 7B-D). Similarly, the blockade of p38 with BIRB796 (p38 inhibitor) inhibited CCL21/CCR7 signalling axis-induced migration of B16F10 cells (Figures 7E and F). However, blocking ERK with PD98059 (ERK inhibitor) failed to inhibit CCL21/CCR7 signalling axis-induced migration of B16F10 cells (Figures 7G and H). These results suggested that JNK and p38 activities played a vital role in the B16F10 cells migration. In summary, the CCL21/CCR7 signalling axis promotes B16F10 cell migration after PTX treatment through activation of JNK and p38.

### CCR7 mAb efficiently abolished lymphatic metastasis of B16F10 tumour cells induced by PTX treatment

**Figure 8:**
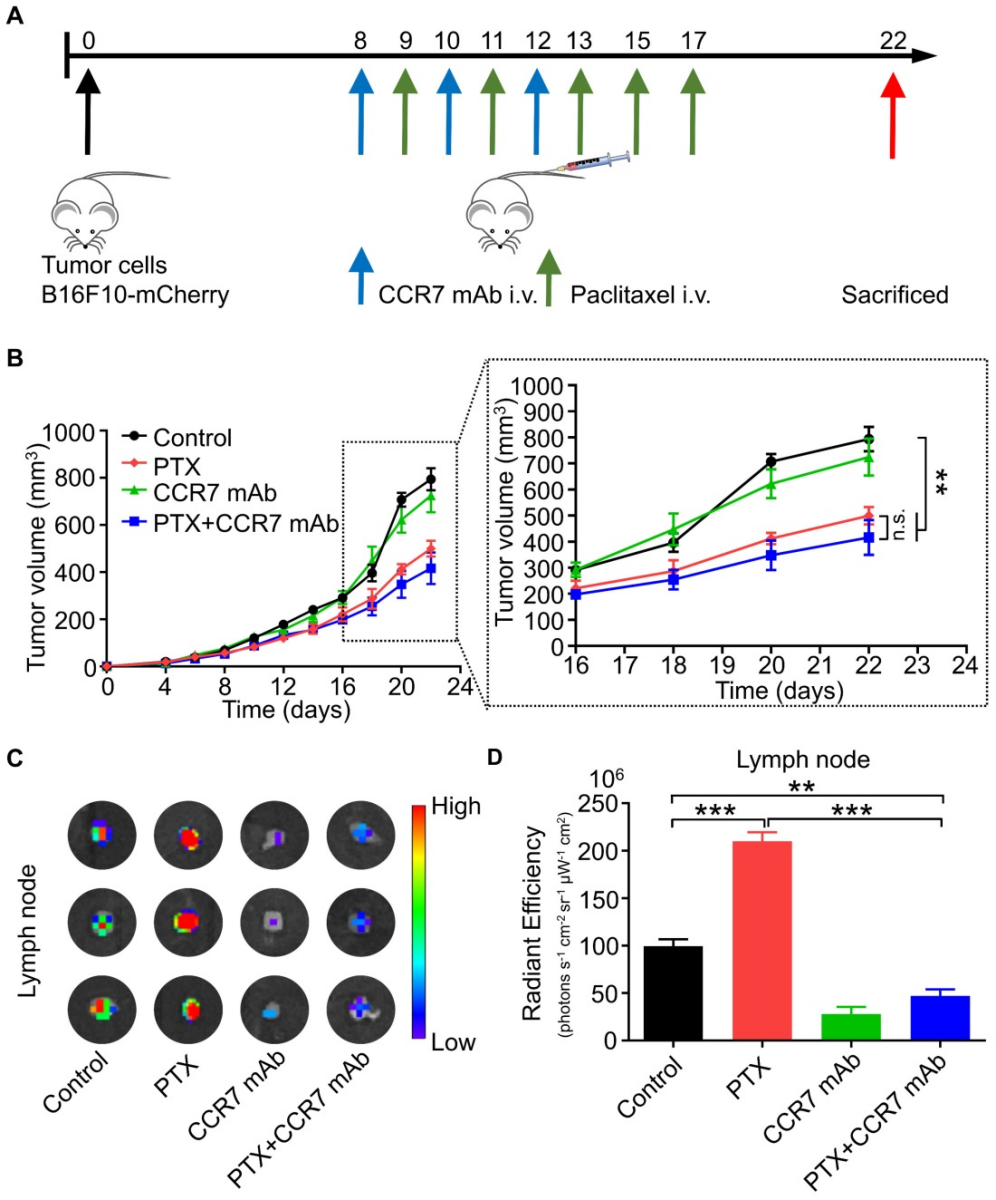
** CCR7 mAb efficiently abolished lymphatic metastasis of B16F10 tumour cells induced by PTX treatment. A.** Experimental design and drug scheduling in tumour-bearing mice.** B.** Tumour growth curve with CCR7 mAb treatments thrice on days 8, 10, and 12 and PTX treatments five times on days 9, 11, 13, 15, and 17 (n = 5 mice/group).** C.** Bioluminescence imaging of the draining LNs with IVIS at the end of the experiment.** D.** The quantification analysis of the bioluminescence signals of the draining LNs. **P < 0.01; ***P < 0.001.

CCR7 mAb was applied to block the CCL21/CCR7 signalling pathway to investigate whether PTX induced lymphatic metastasis was CCR7-dependent *in vivo* 29, 30. The combined therapeutic effect of PTX and CCR7 mAb was tested in the B16F10 melanoma animal model (Figure 8A). A satisfactory tumour growth inhibition effect was observed in the PTX and PTX+CCR7 mAb groups (Figure 8B). Tumours, sentinel lymph nodes, and all major organs were harvested at the endpoint on day 22. Indeed, the lymphatic metastasis to sentinel lymph node was remarkably inhibited in the combination of PTX and CCR7 mAb group than PTX monotherapy group (Figures 8C and D). These results confirmed that blocking the CCL21/CCR7 signalling pathway effectively inhibited the lymphatic metastasis of B16F10 melanoma after PTX treatment. The combination therapy of PTX and CCR7 mAb could simultaneously delay the tumour growth and reduce the lymphatic metastasis of B16F10 melanoma after PTX treatment.

## Discussion

Melanoma metastasises primarily via the lymphatic system, and the extent of lymph node involvement is a key prognostic factor. Upregulation of the CCL21/CCR7 axis results in the migration of tumour cells from primary to lymphatic vessels, promoting lymphatic metastasis of various tumours. Here, we demonstrated that the expression of CCR7 in B16F10 is significantly enhanced after PTX treatment *in vivo* and *in vitro*. PTX increases the migration of B16F10 cells through the enhanced CCR7 expression, resulting in enhanced lymphatic metastasis. Blocking the CCL21/CCR7 axis signalling pathway significantly inhibits the migration of tumour cells treated with PTX. As such, the pro-metastatic effects occurring in response to PTX therapy are mediated, at least in part, by the upregulated expression of CCR7.

Paradoxical effects of chemotherapy on tumour relapse and metastasis promotion were recognised in recent years 31. PTX, a drug that induces mitotic arrest due to activation of the mitotic checkpoint, can promote tumour metastasis 13, 32. Specifically, PTX-induced tumour metastasis is a complex process involving several molecular mechanisms 31. According to a previous report, PTX treatment significantly increased pulmonary and lymphatic metastasis incidence and burden by TLR4-positive tumours 12. TLR4 activation by PTX strongly increased systemic inflammation. Moreover, PTX-mediated activation of TLR4 induced de novo generation of deep intra-tumoural lymphatic vessels that were highly permissive to invasion by malignant cells 12. TLR4 is expressed on both murine and human melanoma cells, and the TLR4 pathway involves melanoma progression and metastasis 33, 34. Cancer cell-derived long pentraxin 3 promoted melanoma migration through TLR4/NF-κB signalling pathway 35. TLR4-knockout melanoma cells exhibited impaired migratory capacity and significantly reduced ability to metastasise to the lungs 36. Suppressing TLR4 or targeting TLR4 in melanomas could inhibit proliferation, migration, and invasion of tumour cells 37, 38. According to previous reports, PTX can induce bone marrow cells with TIE2 receptors to migrate to breast cancer tumours, leading to the formation of the tumour microenvironment of metastasis 4. Moreover, PTX-elicited extracellular vesicles contain high amount of annexin A6 (ANXA6), a Ca^2+^-binding protein, which can promote increased CCL2 expression in endothelial cells and consequent recruitment of inflammatory monocytes expressing CCR2, thereby promoting lung metastasis in breast cancer 5. Furthermore, PTX can increase lymphatic metastasis in breast cancer by promoting autophagy in lymphatic endothelial cells and increasing vessel permeability 13.

The present study reported that PTX could promote lymphatic metastasis of melanoma B16F10. Moreover, tumour tissues exhibited enhanced expression of CCR7 after PTX treatment, which was similar to that observed in B16F10 treated with PTX *in vitro*. Further study suggested that PTX therapy-induced metastasis via enhanced cellular migration regulated these effects by CCR7 expressed in B16F10. However, no significant difference in CCR7 expression was observed in B16F1. This inconsistent phenomenon might be attributed to the heterogeneity of B16F10 and B16F1. Moreover, the expression of CCR7 in tumour tissues of mice treated with PTX (2.5 mg/kg) was increased by 1.3-fold compared with the vehicle control group. Significantly, the upregulation of CCR7 expression in 5 and 10 mg/kg groups was 1.8-fold compared with the vehicle control group, significantly higher than that in the 2.5 mg/kg group. As seen in Figure 1D, lymphatic metastasis was increased dramatically in the PTX (5 and 10 mg/kg) groups but not in PTX 2.5 mg/kg group. This might indicate that CCR7 needs to be increased to a certain level to promote lymphatic metastasis of B16F10 melanoma after PTX treatment. The detailed mechanism remains unclear, which warrants further investigation.

Several studies have indicated that lymphatic metastasis is related to chemokines and chemokine receptors 39. It has been demonstrated that CCL21/CCR7 signalling axis plays a crucial role in the lymphatic metastasis of many tumours. Several studies have reported that lymphatic endothelial cells secrete chemokine CCL21, recruiting tumour cells expressing CCR7 to lymphatic vessels 40, 41. In our studies, the expression of CCR7 was enhanced in B16F10 cells after PTX treatment. Moreover, PTX promoted the migration of B16F10 cells via the CCL21/CCR7 signalling axis. Studies indicated that CCL21/CCR7 signalling axis could induce lymphatic metastasis by stimulating the secretion of vascular endothelial growth factor C (VEGF-C) or D (VEGF-D), leading to lymphangiogenesis 42-44. Furthermore, the cells expressing CCR7 can sense the CCL21 gradient from high to low in the lymphatic vessels and migrate to lymphatic vessels along with a low to the high concentration of CCL21 45, 46. At the molecular level, CCL21/CCR7 can promote lymphatic metastasis of tumours via activating the ERK1/2 22 and JAK2/STAT3 signalling pathways 23. However, the exact mechanism of CCL21/CCR7 signalling axis-induced lymphatic metastasis in melanoma after PTX treatment remains unclear. In the present study, we did not observe increased lymphangiogenesis after PTX treatment *in vivo*, which suggested that PTX-induced lymphatic metastasis may rely on other factors. In the subsequent experiments, we observed that the phosphorylation levels of ERK, JNK, and p38 increased after PTX treatment. The blockade of JNK and p38, a downstream signal of CCL21/CCR7, in PTX-treated tumour cells resulted in a decreased migration of tumour cells. These findings supported our hypothesis that PTX chemotherapy can induce lymphatic tumour metastasis by upregulating the expression of CCR7.

CCL21/CCR7 has been gradually recognised as a potential therapeutic target in many tumours. According to a previous report, several compounds with CCR7-binding ability were screened from more than 2 million combinations by analysing the structure of human CCR7; these compounds have the application potential as CCR7 antagonists to prevent tumour metastasis 47. CCR7-siRNA can inhibit the migration of tumour cells by reducing CCL21/CCR7 binding by inhibiting CCR7 expression at mRNA level 48. Furthermore, targeted delivery of the gene delivery system expressing CCR7 antagonistic proteins to the tumour site significantly inhibited CCL21/CCR7-mediated tumour cell lymphatic migration by blocking the binding of CCL21 to CCR7 in 4T1 and B16F10 30. In addition, CCR7 can be downregulated by topotecan, a semi-synthetic analogue of camptothecin, thereby inhibiting metastasis in breast cancer 49. In this study, PTX treatment could delay tumour growth but increase the risk of tumour metastasis, but inhibition of CCR7 could decrease the PTX-induced migration of B16F10 cells. Targeting the CCL21/CCR7 axis, such as CCR7 neutralising antibody, CCR7-siRNA, CCR7 trap, small molecule antagonist of CCR7, and neutralising antibody of CCL21 in the current preclinical study are likely to enter clinical phase after further study. The CCL21/CCR7 signalling axis might be a superior target compared with other treatment strategies for treating patients with melanoma and lymph node metastasis. However, several questions remain unanswered. It is unclear whether CCL21/CCR7 signalling axis also plays a crucial role in promoting melanoma lymphatic metastasis after other chemotherapeutic drugs. Nevertheless, the combination therapy targeting the CCL21/CCR7 axis and chemotherapy might provide a new direction for melanoma B16F10.

**Figure 9:**
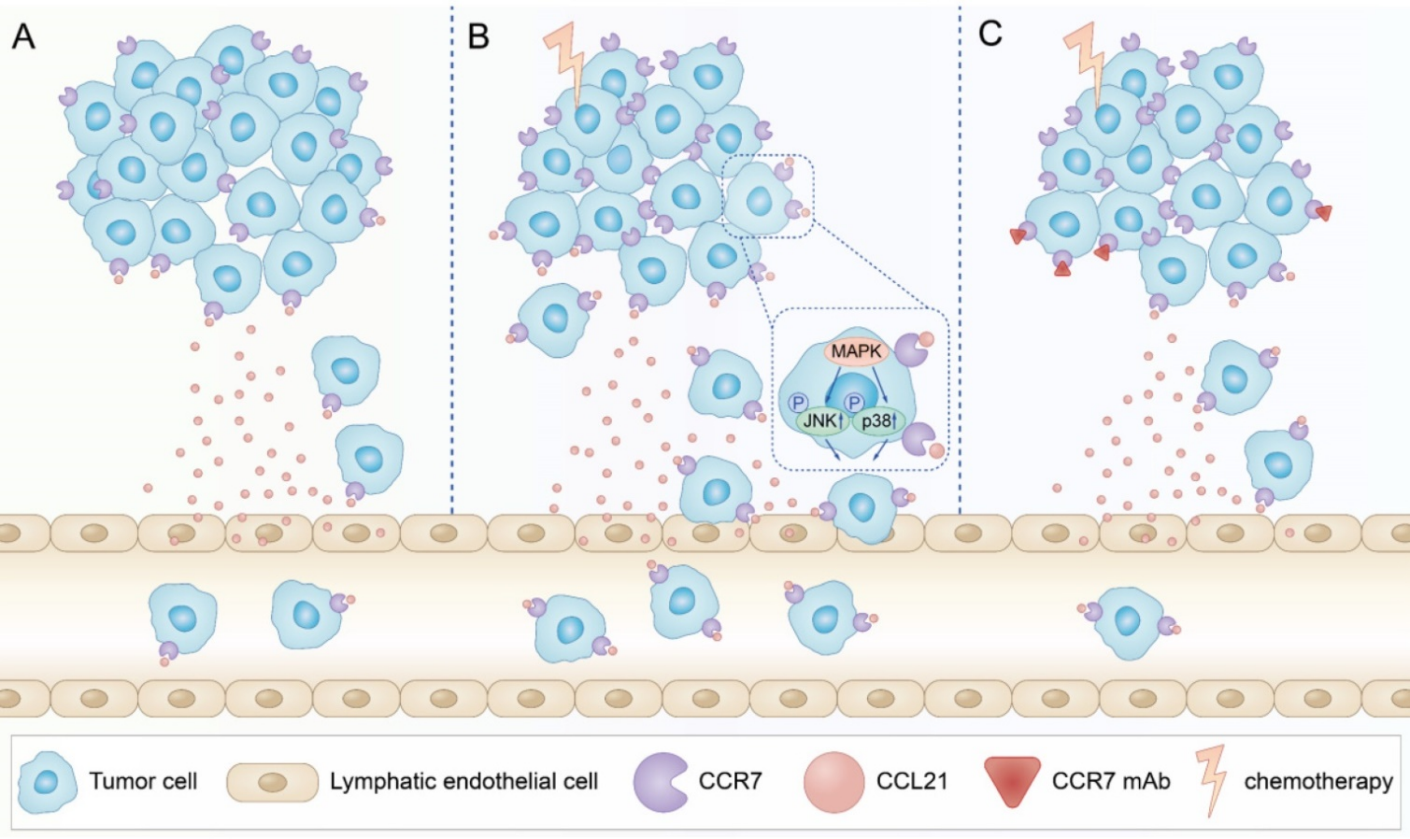
** Schematic illustration of CCL21/CCR7 signalling-mediated lymphatic metastasis in B16F10 cells treated with PTX. A.** Lymphatic metastasis occurred in B16F10 melanoma through CCL21/CCR7 signalling pathway.** B.** PTX treatment enhanced the expression of CCR7 in B16F10 melanoma cells, which facilitated B16F10 cell migration through the activation of JNK and p38 signalling pathways, resulting in enhanced lymphatic metastasis in B16F10 melanoma cells. **C.** Blocking the CCL21/CCR7 signalling pathway through CCR7 mAb efficiently abolished lymphatic metastasis of B16F10 tumour cells induced by PTX treatment.

In summary, our data indicated that PTX treatment could increase lymphatic metastasis of B16F10 melanoma cells upon chemotherapy via the enhancement of the CCL21/CCR7 axis. Further study demonstrated that PTX enhanced the migration of B16F10 cells through activating JNK and p38, the downstream molecules of the CCL21/CCR7 signalling pathway. In turn, downregulating the expression of CCR7 or blocking the CCL21/CCR7 axis ultimately led to the decrease in migration of tumour cells after PTX chemotherapy. The combination therapy of PTX and CCR7 mAb could simultaneously delay the tumour growth and reduce the lymphatic metastasis of B16F10 melanoma after PTX treatment (Figure 9). Thus, inhibition of the CCL21/CCR7 axis may be used to counteract chemotherapy-induced metastasis, providing a novel strategy to improve chemotherapeutic treatment for melanoma.

## Supplementary Material

Supplementary figures and tables.

## Abbreviations

PTX: paclitaxel
CCL21: C-C motif chemokine ligand 21
CCR7: C-C chemokine receptor type 7
DC: dendritic cells
VEGF-C: vascular endothelial growth factor C
VEGF-D: vascular endothelial growth factor D
TLR4: toll-like receptor-4
MAPK: mitogen-activated protein kinase
DDP: cisplatin
DOX: doxorubicin
