# Supplementary material for: Paclitaxel treatment enhances lymphatic metastasis of B16F10 melanoma cells via CCL21/CCR7 axis
Source: Int J Biol Sci. 2022 Jan 24;18(4):1476–90. doi: 10.7150/ijbs.67138 (PMC8898365; doi:10.7150/ijbs.67138)
Supplement: Supplementary file 1 — Supplementary figures and tables. [file ijbsv18p1476s1.pdf]

Paclitaxel treatment enhances lymphatic metastasis of B16F10 melanoma cells via CCL21/CCR7 axis

Li Zhang[1,2 †], Linyu Zhu[1 †], Xiaohan Yao[1], Xiaohan Lou[1], Jiajia, Wan[1], Xixi Duan[1], Longze Pan[1], Anqi Li[1], Zhuoyu Gu[1], Ming Wang[1], Fazhan Wang[1]*, Zhihai Qin[1,2,3]*

[1]Medical Research Center, The First Affiliated Hospital of Zhengzhou University, Zhengzhou University, Zhengzhou, 450052, Henan, China

[2]Academy of Medical Sciences, Zhengzhou University, Zhengzhou, 450052, Henan, China

[3]Institute of Biophysics, Chinese Academy of Sciences, Beijing 100101, China

[†] These authors contributed equally to this article.

***Corresponding author**

Address: Medical Research Center, The First Affiliated Hospital of Zhengzhou University, No. 1, Jianshe East Road, Zhengzhou, 450052, Henan, China.

Telephone: + 86-371-66913632

Fax: + 86-371-66913632

E-mail: zhihai@ibp.ac.cn (Zhihai Qin), fazhanwang_20@zzu.edu.cn (Fazhan Wang)

Supplementary materials

Supplementary figures

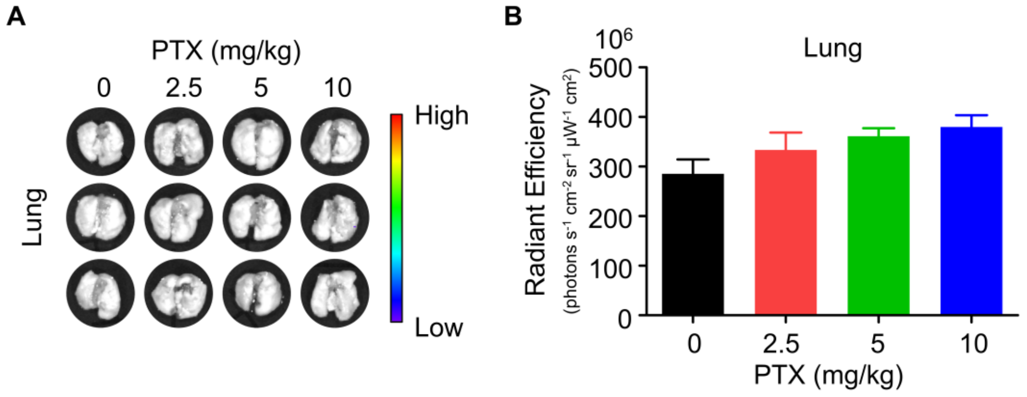

Figure S1. PTX had no effect on lung metastasis in B16F10-mCherry-bearing mice. A. Bioluminescence imaging of the lung at the endpoint. **B.** The quantification analysis of the bioluminescence signals of the lung.

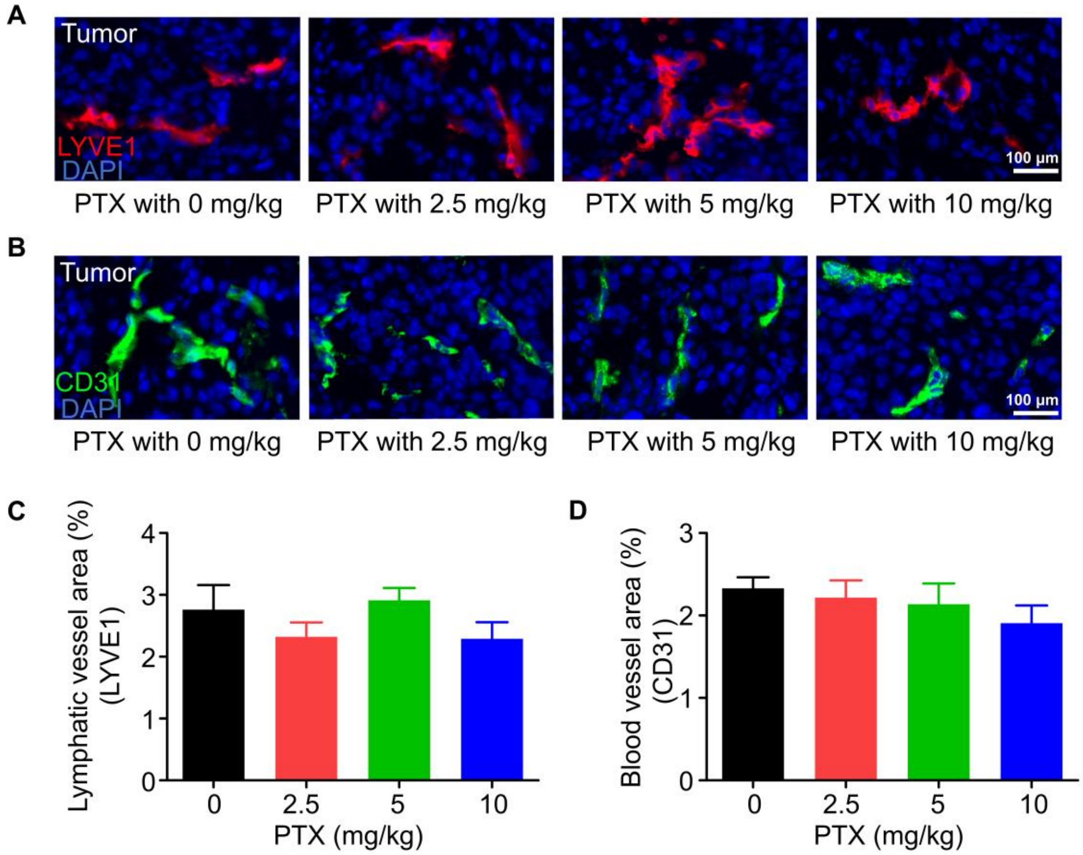

Figure S2. PTX did not influence the relative expression of LYVE1 and CD31. A. Immunofluorescence staining of lymphatic vessels marker LYVE1 in tumour tissues (Scale bar: 100 μm, red: LYVE1, blue: nucleus). **B.** Immunofluorescence staining of blood vessels marker CD31 in tumour tissues (Scale bar: 100 μm, green: CD31, blue: nucleus). **C.** The quantification of lymphatic vessels marker LYVE1. **D.** The quantification of blood vessels marker CD31.

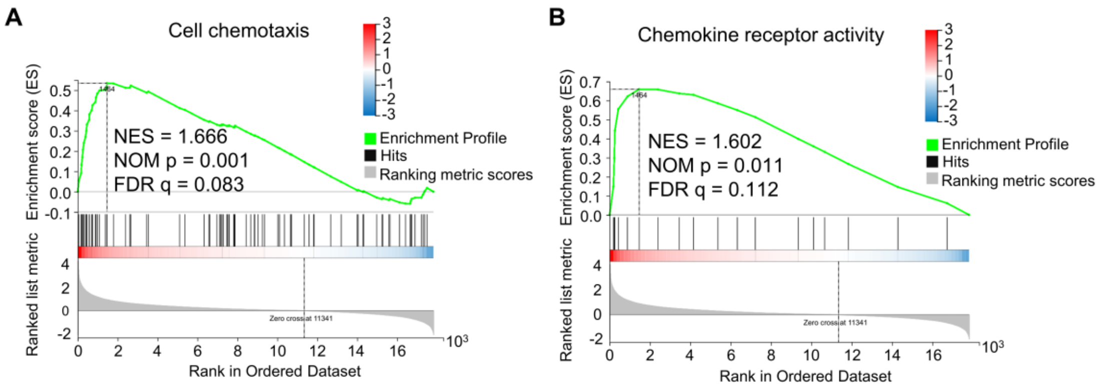

Figure S3. Plot of gene set enrichment analysis (GESA) on B16F10 cells related to cell chemotaxis and chemokine receptor activity. A–B. Plot of GESA using RNA-seq expression profile of B16F10 cells with and without PTX treatment (GO: 0060326, cell chemotaxis; GO: 0004950, chemokine receptor activity).

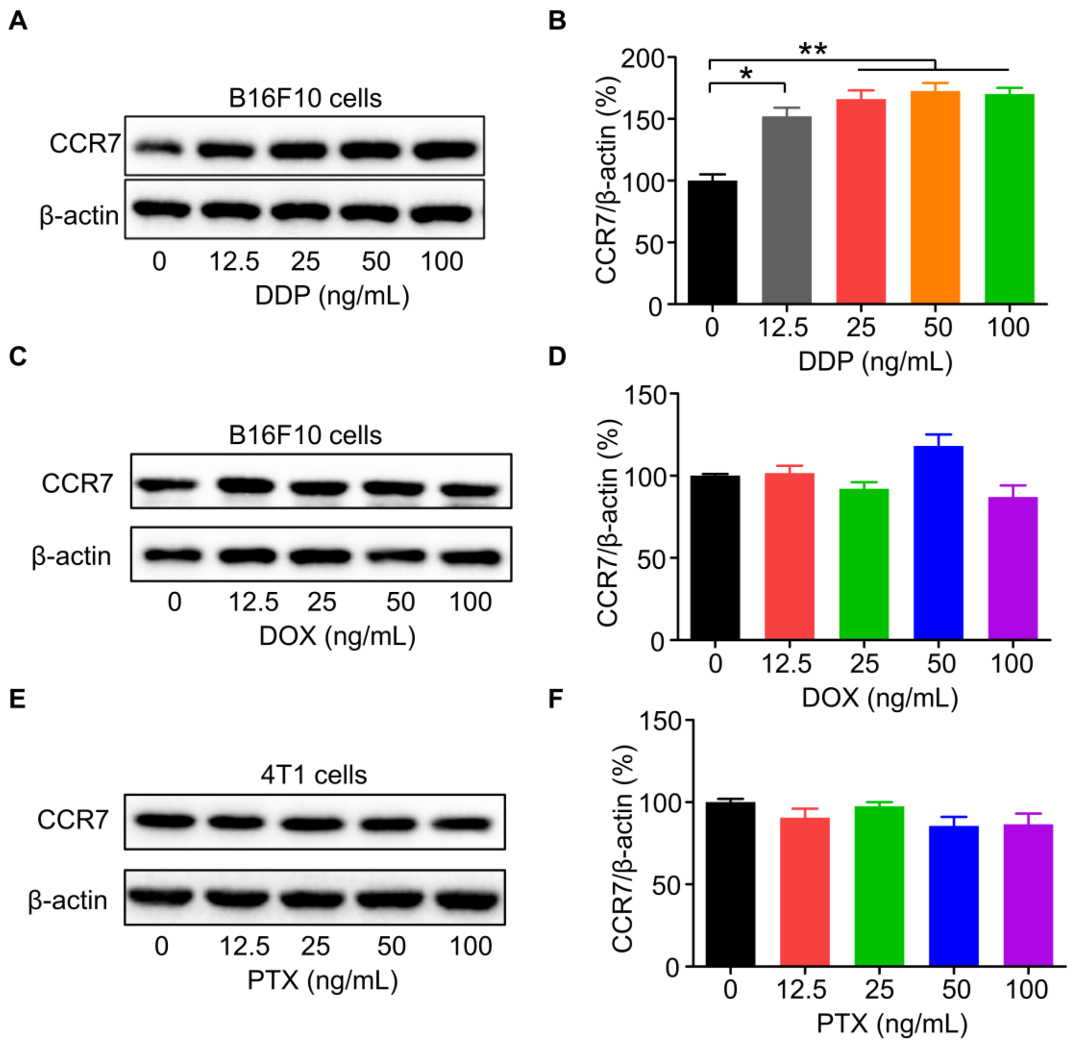

Figure S4. DDP upregulated the expression of CCR7 in B16F10 cells.

A. Western blot of CCR7 in B16F10 cells with DDP treatment. **B**. The quantification of western blot of CCR7 in B16F10 cells with DDP treatment. **C.** Western blot of CCR7 in B16F10 cells with DOX treatment. **D**. The quantification of western blot of CCR7 in B16F10 cells with DOX treatment. **E.** Western blot of CCR7 in 4T1 cells with PTX treatment. **F**. The quantification of western blot of CCR7 in 4T1 cells with PTX treatment. $*P < 0.05$; $**P < 0.01$.

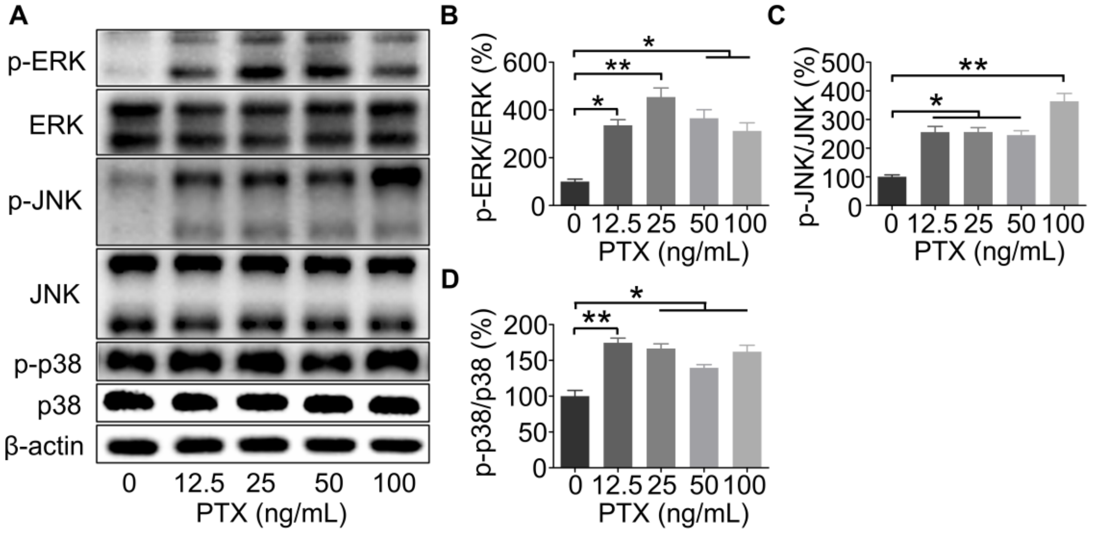

Figure S5. The phosphorylation level of ERK, JNK, and p38 in B16F10 cells 12 h after PTX treatment was enhanced. A. Phosphorylation of ERK, JNK, and p38 in B16F10 cells 12 h after PTX treatment was assessed with western blotting. **B.** The quantification of phosphorylation of ERK in B16F10 cells 12 h after PTX treatment. **C.** The quantification of phosphorylation of JNK in B16F10 cells 12 h after PTX treatment. **D.** The quantification of phosphorylation of p38 in B16F10 cells 12 h after PTX treatment. *P < 0.05; **P < 0.01.

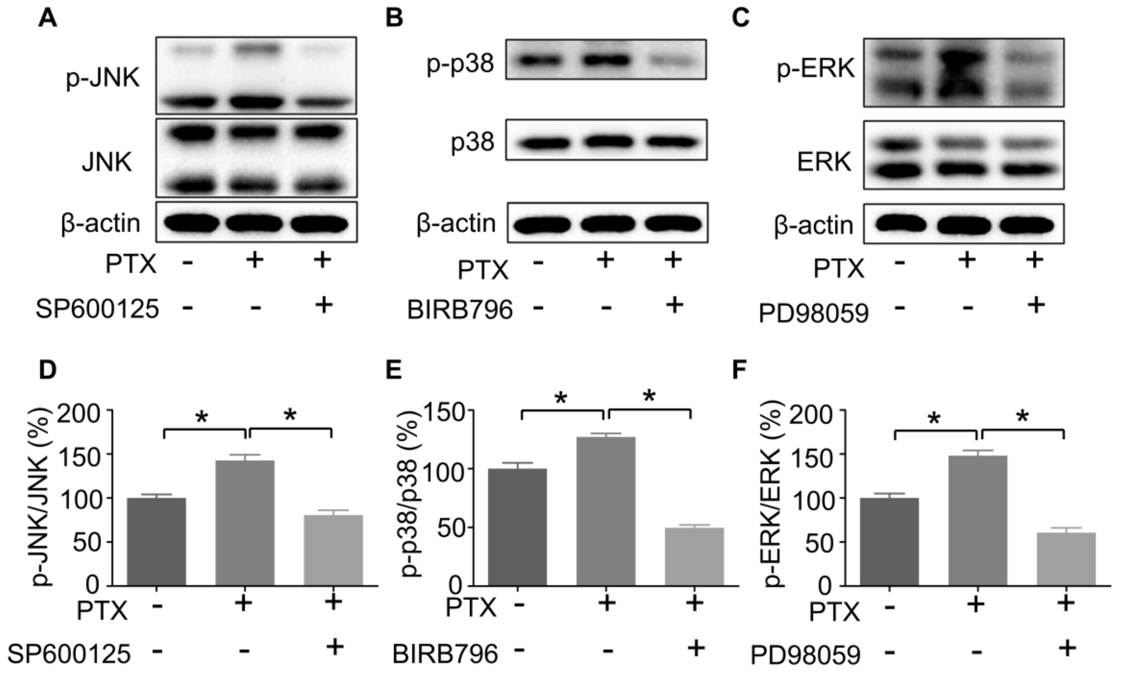

Figure S6. Blockade of JNK, p38, and ERK by SP600125, BIRB796, and PD98059. A. Western blot of blockade of JNK by SP600125 (JNK inhibitor). **B.** Western blot of blockade of p38 by BIRB796 (p38 inhibitor). **C.** Western blot of blockade of ERK by PD98059 (ERK inhibitor). **D.** The quantification of western blot of blockade of JNK. **E.** The quantification of western blot of blockade of p38. **F.** The quantification of western blot of blockade of ERK. $^*P < 0.05$.

Supplementary tables

Table S1. Details of antibodies

Antibodies	Company	Lot No.
Rabbit anti-CCR7	ABclonal	A0121
Rabbit anti-ERK	Cell Signaling Technology	4695
Rabbit anti-phospho-ERK	Cell Signaling Technology	4370
Rabbit anti-JNK	Cell Signaling Technology	9258
Rabbit anti-phospho-JNK	Cell Signaling Technology	4668
Rabbit anti-p38	Cell Signaling Technology	8690
Rabbit anti-phospho-p38	Cell Signaling Technology	4511
Rabbit anti-β-actin	ABclonal	AC038
HRP goat anti-rabbit IgG H+L	ABclonal	AS014
Rabbit anti-CCR7	Abcam	ab32527
Rabbit anti-LYVE1	Abcam	ab14917
Rat anti-mouse CD31	BD	550274
Alexa Fluor™ 555 F (ab') 2 fragment of goat anti-rabbit IgG (H+L)	Invitrogen	A21430
Alexa Fluor™ 488 donkey anti-rat IgG (H+L)	Invitrogen	A21208

Table S2. Sequences of siRNA

Gene name	sense（5'-3'）	antisense（5'-3'）
Ccr7-Mus-467	GGGCAUCUUUGGCAUCUAUTT	AUAGAUGCCAAAGAUGCCCTT
Ccr7-Mus-796	GUUUCUGCUACCUCAUUAUTT	AUAAUGAGGUAGCAGAAACTT
Ccr7-Mus-939	GUGGCCAACUUCAACAUCATT	UGAUGUUGAAGUUGGCCACTT
Mouse GAPDH-420	CACUCAAGAUUGUCAGCAATT	UUGCUGACAAUCUUGAGUGAG
Negative control FAM	UUCUCCGAACGUGUCACGUTT	ACGUGACACGUUCGGAGAATT
Negative control	UUCUCCGAACGUGUCACGUTT	ACGUGACACGUUCGGAGAATT

Table S3. Primers used for qRT-PCR

Species	Gene name	Primer sequence（5'-3'）
Mouse	CCR7	F: TCCTTCTCATCAGCAAGCTGT<br>R: GAGGCAGCCCAGGTCCTTGAA
Mouse	β-actin	F: ATGCCCTGAGGCTCTTTTCC<br>R: TGCTAGGAGCCAGAGCAGTA
